# Nutrient Availability and Phage Exposure Alter the Quorum-Sensing and CRISPR-Cas-Controlled Population Dynamics of Pseudomonas aeruginosa

**DOI:** 10.1128/msystems.00092-22

**Published:** 2022-06-14

**Authors:** Stephen Dela Ahator, Sadhanna Sagar, Minya Zhu, Jianhe Wang, Lian-Hui Zhang

**Affiliations:** a Guangdong Province Key Laboratory of Microbial Signals and Disease Control, Integrative Microbiology Research Centre, South China Agricultural University, Guangzhou, China; b Guangdong Laboratory for Lingnan Modern Agriculture, South China Agricultural University, Guangzhou, China; c Centre for New Antibacterial Strategies (CANS) & Research Group for Host-Microbe Interactions, Faculty of Health Sciences, The Arctic University of Norway, Tromsø, Norway; University of Pretoria

**Keywords:** quorum sensing, CRISPR-Cas system, population dynamics, phage infection

## Abstract

Quorum sensing (QS) coordinates bacterial communication and cooperation essential for virulence and dominance in polymicrobial settings. QS also regulates the CRISPR-Cas system for targeted defense against parasitic genomes from phages and horizontal gene transfer. Although the QS and CRISPR-Cas systems are vital for bacterial survival, they undergo frequent selection in response to biotic and abiotic factors. Using the opportunistic Pseudomonas aeruginosa with well-established QS and CRISPR-Cas systems, we show how the social interactions between the acyl-homoserine lactone (AHL)-QS signal-blind mutants (Δ*lasRrhlR*) and the CRISPR-Cas mutants are affected by phage exposure and nutrient availability. We demonstrate that media conditions and phage exposure alter the resistance and relative fitness of Δ*lasRrhlR* and CRISPR-Cas mutants while tipping the fitness advantage in favor of the QS signal-blind mutants under nutrient-limiting conditions. We also show that the AHL signal-blind mutants are less selected by phages under QS-inducing conditions than the CRISPR-Cas mutants, whereas the mixed population of the CRISPR-Cas and AHL signal-blind mutants reduce phage infectivity, which can improve survival during phage exposure. Our data reveal that phage exposure and nutrient availability reshape the population dynamics between the Δ*lasRrhlR* QS mutants and CRISPR-Cas mutants, with key indications for cooperation and conflict between the strains.

**IMPORTANCE** The increase in antimicrobial resistance has created the need for alternative interventions such as phage therapy. However, as previously observed with antimicrobial resistance, phage therapy will not be effective if bacteria evolve resistance and persist in the presence of the phages. The QS is commonly known as an arsenal for bacteria communication, virulence, and regulation of the phage defense mechanism, the CRISPR-Cas system. The QS and CRISPR-Cas systems are widespread in bacteria. However, they are known to evolve rapidly under the influence of biotic and abiotic factors in the bacterial environment, resulting in alteration in bacterial genotypes, which enhance phage resistance and fitness. We believe that adequate knowledge of the influence of environmental factors on the bacterial community lifestyle and phage defense mechanisms driven by the QS and CRISPR-Cas system is necessary for developing effective phage therapy.

## INTRODUCTION

Pseudomonas aeruginosa is a ubiquitous opportunistic pathogen with an intricate regulatory system enabling it to thrive in myriads of stressful environmental conditions. It causes diseases in immunocompromised individuals and frequently colonizes the lungs of cystic fibrosis (CF) patients (1). Successful colonization and establishment of infection by P. aeruginosa involve coordinated expression of virulence genes and factors in concert with the population density via releasing and sensing diffusible molecules (2–4). The release and response to chemical signals and cell density, known as quorum sensing (QS), controls traits such as biofilm formation and production of virulence arsenals crucial for host tissue damage, systemic invasion, and evasion from the host immune factors (5). In P. aeruginosa, QS consists of three canonical systems, *las*, *pqs*, and *rhl*, which are hierarchically organized (6, 7). Also, a fourth signal known as IQS [2-(2-hydroxyphenyl)-thiazole-4-carbaldehyde], which is a *las*-independent regulator of the *pqs* and *rhl* systems under phosphate stress, was identified with an unknown receptor (8). The two acyl-homoserine lactone (AHL) QS systems, the *las* and *rhl* systems, are composed, respectively, of the *lasR*-*lasI* and the *rhlR*-*rhlI* genes, which share some overlaps in their regulon (9). The LasI and RhlI catalyze the synthesis of *N*-3-oxo-dodecanoyl homoserine lactone (3OC12-HSL) and butyryl-HSL (C4-HSL), which interact with their respective transcriptional regulators, LasR and RhlR. The *las* system regulates the production of virulence factors such as proteases and elastase. Also, the *rhl* system regulates the production of compounds such as pyocyanin and cyanide (9, 10).

The cell-cell communication established by the QS system enables cooperative behavior between bacteria where they collectively regulate the production of factors required for their survival in response to biotic and abiotic stress (11–13). Production of virulence factors such as protease and siderophores via QS underlies a cooperative activity or community lifestyle which benefits other bacteria in the population (14–16). Evolution theory backed by experimental evidence has shown that cooperative behavior is exploited by cheats that evolve in the population due to selective pressure. These cheats avoid the cost of producing QS-regulated factors but benefit from the public goods released by others. The cheats either produce the QS signal but do not increase the response for exoproduct production (signal blind) or do not produce the QS signal but can monitor the local population density (signal negative) (17, 18). In P. aeruginosa, the *lasR* and *rhlR* mutants (signal blind) are most commonly isolated during infection, as they gain a competitive advantage by avoiding the expression of QS-regulated genes and exploiting extracellular factors produced by the other strains in the population (18–20). Conversely, the signal-negative mutants with defective *lasI* and *rhlI* respond to QS signals from surrounding cells to activate the expression of QS-controlled genes and produce extracellular factors used by the cheats (12). The evolution of the cheating population is associated with increased fitness advantage, adaptability, and persistence of P. aeruginosa isolates under various stress conditions.

Although QS controls the reduction of phage receptors and increases resistance to phage infection (21–23), the high population density predisposes bacteria to phage infections, as it imposes a spatial vulnerability for phage interaction (24–27). In safeguarding against the deleterious effect of phage infection, bacteria possess various defense mechanisms such as the clustered regularly interspaced short palindromic repeats (CRISPR)and CRISPR-associated proteins (CRISPR-Cas) system, which provides adaptive heritable immunity against foreign nucleic acids from phages and plasmids. The CRISPR system is composed of a repetitive array of sequences complementary to parasitic genomes. The CRISPR array is transcribed into CRISPR RNAs (crRNAs), which, in association with the Cas proteins, target complementary invading sequences and mediate their cleavage (28–30). In effect, this averts the harmful impact of phage infection or horizontal gene transfer (HGT) (31).

The expression and maintenance of the CRISPR-Cas system are metabolically costly and are dependent on a combination of the biotic and abiotic factors encountered by the bacteria (32, 33). Nutrient availability, metabolic stress, parasite exposure, and cell density influence the effectiveness of the CRISPR-Cas system in defending against phage infection (26, 27, 34). Regulation of the CRISPR-Cas system is integrated into QS and presents a robust defense mechanism against phage infection at high cell density where the bacteria is most vulnerable. In P. aeruginosa, the AHL (*las* and *rhl*)-QS system regulates the CRISPR-Cas gene expression and HGT interference (27).

As observed with the evolution of QS mutants, clinical isolates with defective or lacking CRISPR-Cas systems have been reported (35–37). The selection of the CRISPR-Cas system is linked to the trade-off associated with its fitness cost and maintenance in the absence of phages (32) and other metabolic functions in the cell (34). At high phage exposure, the expression of the CRISPR-Cas system is not metabolically beneficial, and thus, alternative defense mechanisms like surface modification are preferable (32, 34). Also, in the absence of phages, selection against the CRISPR-Cas system can be beneficial in preventing autoimmunity (38, 39) or allowing the acquisition of beneficial adaptive traits encoded by foreign genetic elements (40). These imply that the associated cost of CRISPR-Cas expression and maintenance is dependent on its functions in the cell and the level of phage exposure (32, 34).

Due to the increasing interest in phage therapy as antibiotic resistance rapidly rises, understanding the coevolutionary outcomes of phages on polymicrobial infections is vital for developing effective phage therapy systems. Both phage exposure and resource availability remain inevitable stress factors encountered by bacteria in their natural environments, which influence bacterial sociomicrobiology and evolution (11, 12, 20, 34, 41, 42). P. aeruginosa infections in humans are composed of genetically varying cells exposed to a repertoire of phages (15, 43–48). The QS and CRISPR-Cas systems play key roles in maintaining the population dynamics during infection and defending against phage infections. However, the two systems evolve rapidly under biotic and abiotic stress in the environment (16, 49). Despite the increasing knowledge of the QS and CRISPR-Cas systems, the social interactions between the two systems in P. aeruginosa during phage exposure and growth in nutrient-rich and nutrient-limited conditions remain unclear.

Here, we investigate the intraspecific relationship between the QS and CRISPR-Cas systems and their impact on P. aeruginosa social lifestyle when exposed to stress factors such as phage exposure and nutrient availability. By exposing P. aeruginosa strains composed of AHL signal-blind and CRISPR-Cas mutants to these biotic and abiotic factors, we show the impact of both systems on the fitness of the bacteria during phage exposure. From this study, we further gain insight into the cooperation and conflict occurring in the microbial community due to phage exposure and nutrient availability, where we highlight the influence of both factors on the alteration of the bacteria community lifestyle.

## RESULTS

### Phage effects on the signal-blind (Δ*lasRrhlR)* and CRISPR mutants under QS-inducing and -noninducing conditions.

We investigated the impact of QS and the CRISPR-Cas system on the social behavior, phage resistance, and fitness of P. aeruginosa when exposed to phages under different nutrient sources. For this study, we used the P. aeruginosa UCBPP-PA14 strain (PA14) possessing the type I-F CRISPR-Cas system as the wild type (WT) and its derivatives, PA14 AHL signal-blind cheats (Δ*lasRrhlR*) and the CRISPR-Cas mutant (ΔCRISPR) with the 6 Cas genes and 2 CRISPR arrays deleted. The M9 media supplemented with Casamino Acids (M9-CAA) and casein (M9-casein) as sole carbon sources were used as growth conditions to examine the impact of phages Pf4 and ZCO1 on the social behavior of the Δ*lasRrhlR* and ΔCRISPR strains. The M9-casein, which induces QS behavior and promotes social cheating in AHL signal-blind strains (19, 20), was used to investigate the impact of QS on the sociomicrobiology of the bacteria under the conditions tested in this study. The M9-casein and M9-CAA both serve as nutrient-limiting conditions; however, M9-CAA does not induce QS behavior and social cheating (12).

**FIG 1:**
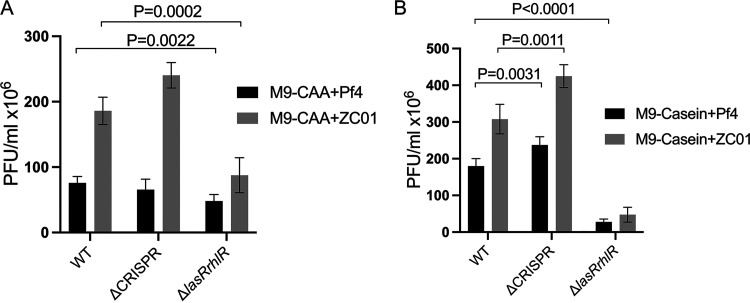
Medium conditions influence phage infection capacity in P. aeruginosa strains. The Pf4 and ZCO1 phage density were measured in the WT, ΔCRISPR, and Δ*lasRrhlR* strains grown in M9-CAA (A) and M9-casein (B) after 16 h of phage addition. The Pf4 and ZCO1 phages were added at the beginning of the experiment at a multiplicity of infection (MOI) of 0.001. All experiments were repeated at least 5 times. Error bars represent ± SD. A *P* value of <0.05 is considered significant.

In the monocultures using M9-CAA and M9-casein media, we tested the infection capacity of Pf4 and ZCO1 phages on the WT, ΔCRISPR, and Δ*lasRrhlR* strains. We observed that the QS media (M9-casein) stimulated higher phage accumulation than the M9-CAA. The ZCO1 phage showed a greater infection capacity in both M9-casein and M9-CAA media than Pf4 in the WT and ΔCRISPR strains (Fig. 1A and B). Except for Pf4 infection capacity in M9-CAA, the phage infection capacity in the ΔCRISPR strains was higher than the WT (Fig. 1A and B; see Fig. S1A and B in the supplemental material) (WT/ΔCRISPR in M9-CAA and ZCO1, *P* = 0.0011). These show that the CRISPR-Cas system plays a role in defense against both phages in the QS media (M9-casein) and only ZCO1 in the M9-CAA. It can also imply that the CRISPR-Cas defense against the phages is influenced by the media conditions. Surprisingly, significantly reduced phage densities were observed in the Δ*lasRrhlR* strains compared to both the WT and ΔCRISPR strains when exposed to ZCO1 in the M9-casein and M9-CAA media (Fig. 1A and B; Fig. S1A and B). In M9-casein specifically, the Δ*lasRrhlR* strains exhibit increased resistance to the Pf4 and ZCO1 phages compared to the WT and ΔCRISPR strains (Fig. 1B). A defective QS system impairs growth of P. aeruginosa in M9-casein (Fig. S2), which can influence the phage infection capacity of Δ*lasRrhlR* strains. This is in agreement with previous reports indicating that a reduction in cell growth rate decreases phage infection and propagation (50, 51).

10.1128/msystems.00092-22.2 FIG S1 The cell density of the WT, ΔCRISPR, and Δ*lasRrhlR* strains following 16-hour infection with phages ZCO1 and Pf4 in M9-CAA (A) and M9-casein (B). The Pf4 and ZCO1 phages were added at the beginning of the experiment at an MOI of 0.001. The bacterial densities indicated in CFU/mL were measured by spread plating serial dilutions of the culture on LB agar. All experiments were repeated at least 5 times. Error bars represent ± SD. A *P* value of <0.05 is considered significant. Download FIG S1, TIF file, 2.1 MB. Copyright © 2022 Ahator et al. 2022 Ahator et al. https://creativecommons.org/licenses/by/4.0/ This content is distributed under the terms of the Creative Commons Attribution 4.0 International license.

10.1128/msystems.00092-22.3 FIG S2 Growth curve of P. aeruginosa strains grown in LB, M9-casein, and M9-CAA. Download FIG S2, TIF file, 2.1 MB. Copyright © 2022 Ahator et al. 2022 Ahator et al. https://creativecommons.org/licenses/by/4.0/ This content is distributed under the terms of the Creative Commons Attribution 4.0 International license.

### Phage exposure influences the fitness of P. aeruginosa isolates.

The regulation of QS-associated factors in P. aeruginosa is metabolically demanding and imposes a fitness cost (11, 20, 52). Consequently, QS-defective mutants such as the signal-blind QS mutants (*lasR* and *rhlR*) are frequently identified among P. aeruginosa clinical isolates and exhibit increased fitness advantage by exploiting the QS-proficient strains (19, 20). Likewise, besides reports of CRISPR-Cas-defective isolates, there is evidence supporting the fitness cost associated with maintenance of the CRISPR-Cas-mediated defense in the absence of phages and under certain environmental conditions (32, 34–37). Hence, we decided to investigate the fitness cost associated with loss of the QS and CRISPR-Cas systems during coculture of the strains in M9-casein or M9-CAA media with and without the phages. We determined the relative fitness of the strains by examining the increase in the frequency of one bacterial strain over another. Also, the strains were marked with in *trans* expression constructs of enhanced green fluorescent protein (EGFP), mCherry, and LacZ for the competition assay. We used these constructs since they did not impose any growth defects when expressed in the WT, Δ*lasRrhlR*, and ΔCRISPR strains (Fig. S3).

10.1128/msystems.00092-22.4 FIG S3 Growth curve of P. aeruginosa strains in LB expressing mCherry, EGFP, and LacZ in *trans* from the pUCP19 vector backbone. Download FIG S3, TIF file, 2.1 MB. Copyright © 2022 Ahator et al. 2022 Ahator et al. https://creativecommons.org/licenses/by/4.0/ This content is distributed under the terms of the Creative Commons Attribution 4.0 International license.

**FIG 2:**
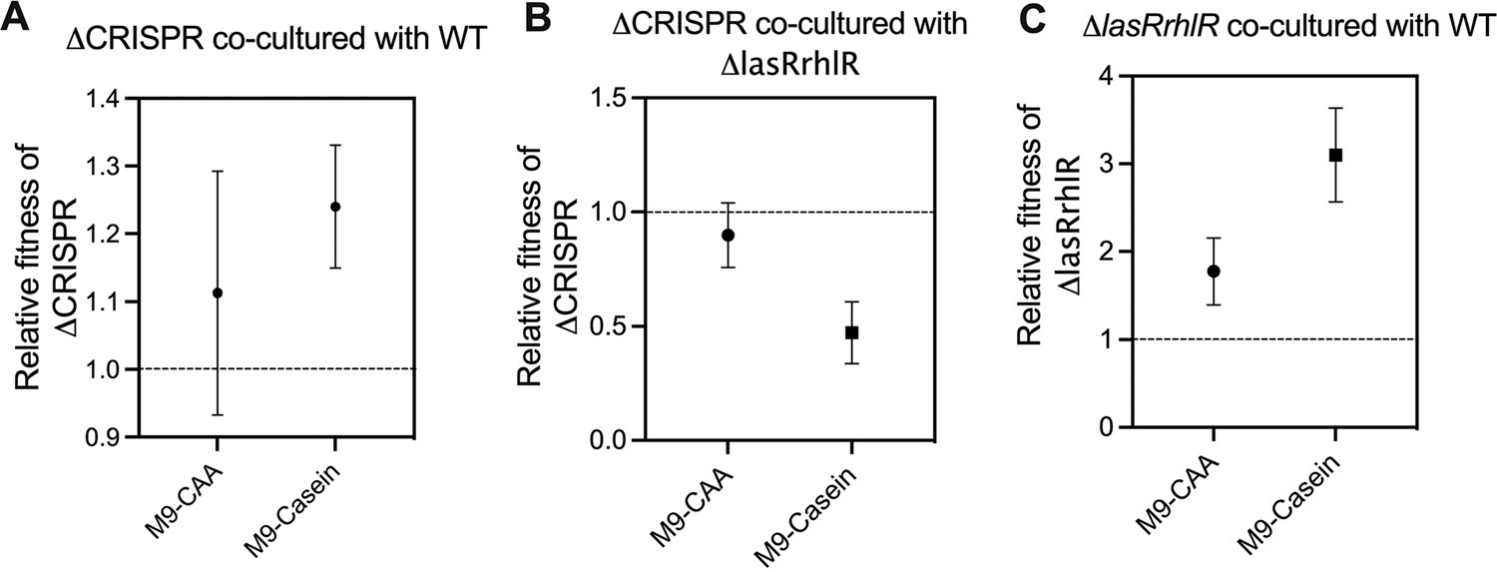
Effect of media conditions on the relative fitness of P. aeruginosa strains. (A and B) Relative fitness of the ΔCRISPR strains grown in coculture with the WT and Δ*lasRrhlR* strains, respectively, in M9-CAA and M9-casein. (C) Relative fitness of the Δ*lasRrhlR* strains grown in coculture with the WT in M9-CAA and M9-casein media. All experiments were repeated at least 5 times.

In the absence of phages, the ΔCRISPR strain showed an increased fitness advantage over the WT in M9-casein (Fig. 2A) (ΔCRISPR/WT in M9-casein, *P* = 0.00812) but exhibited reduced fitness when cultured with Δ*lasRrhlR* in M9-casein (Fig. 2B) (ΔCRISPR/Δ*lasRrhlR* in M9-casein, *P* = 0.0039). During coculture in M9-CAA, the ΔCRISPR strain showed similar fitness against the WT and Δ*lasRrhlR* strains (Fig. 2A and B). As expected and in agreement with previous work, the Δ*lasRrhlR* strain exhibited increased fitness over the WT in M9-casein (*P* = 0.0043) and M9-CAA (*P* = 0.0262) (Fig. 2C) (12, 19, 20).

**FIG 3:**
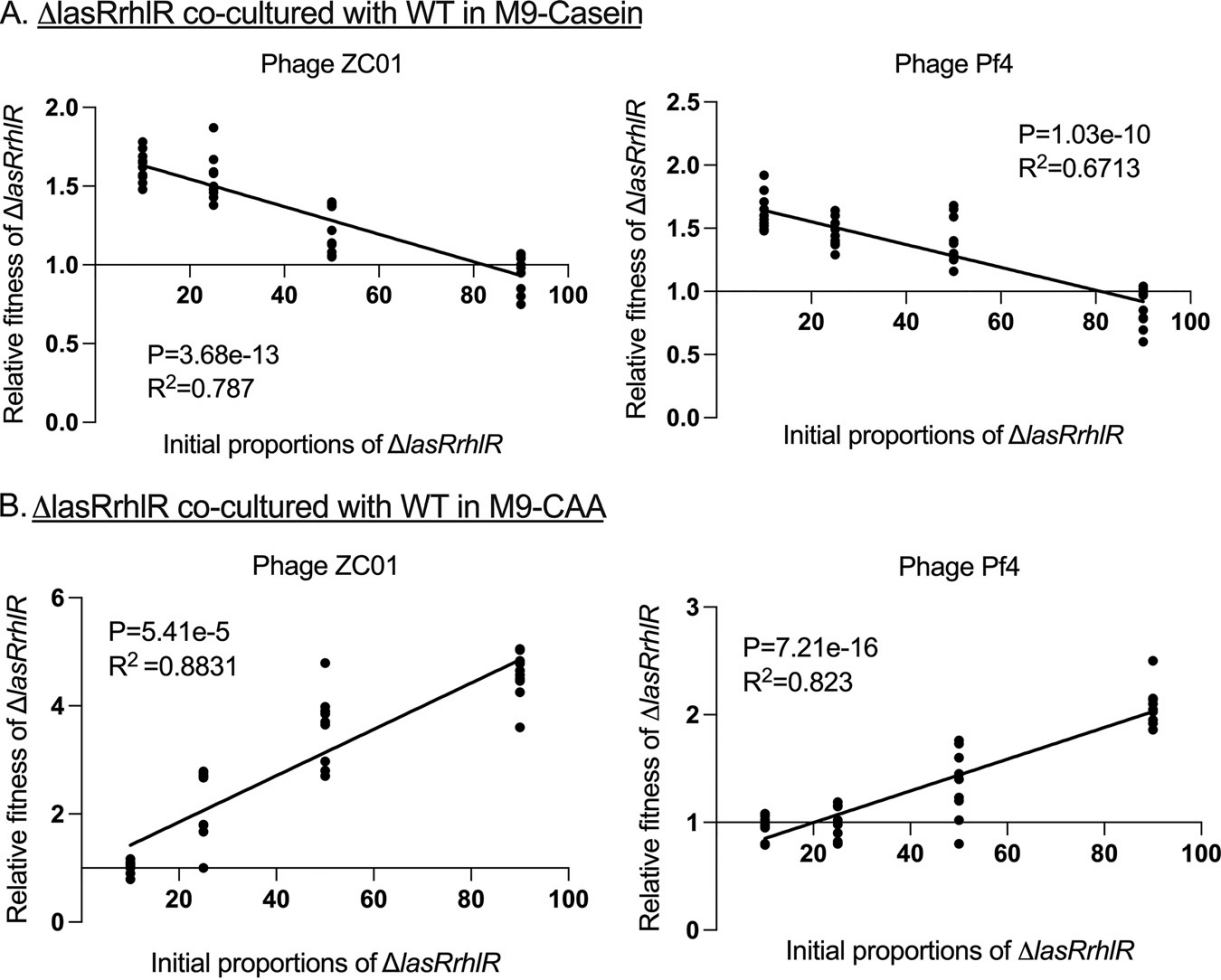
Frequency-dependent relative fitness of AHL signal-blind mutants when exposed to phages. The relative fitness of Δ*lasRrhlR* strains cocultured with WT in M9-casein (A) and in M9-CAA (B) with the Pf4 and ZCO1 phages. The strains were cocultured for 24 h with their relative initial proportions as 10%, 50%, 75%, and 90% of the population. The goodness of fit is indicated by *R*^2^ and was analyzed using analysis of variance (ANOVA) with *P* values indicated (*n* = 10 biologically independent samples for each initial proportion of strains).

We further investigated the frequency-dependent relative fitness of the WT, Δ*lasRrhlR*, and ΔCRISPR strains during ZCO1 and Pf4 exposure by coculturing in M9-casein and M9-CAA for 24 h. In M9-casein, the Δ*lasRrhlR* strains showed a negative frequency-dependent fitness, where their relative fitness decreased with increasing initial proportion in the population (Fig. 3A). This is in agreement with social evolution theory where the fitness of the cheaters decreases with their increasing frequency in the population due to fewer cooperators to exploit (12, 53). It further highlights the ability of the Δ*lasRrhlR* strains to exploit the WT for resources while enjoying the benefit of phage resistance as long as the WT population is high enough to supply the threshold public resources. In M9-CAA, where QS-regulated factors are not required for growth, the relative fitness of the Δ*lasRrhlR* increased as their initial proportion increased in the population (Fig. 3B). However, here, we observed that when Δ*lasRrhlR* strains made up 10% of the initial mixture, they showed similar dominance as the WT when exposed to the phages (Fig. 3B), which can be attributed to the increased resistance of the Δ*lasRrhlR* strains to both phages compared to the WT in M9-CAA (Fig. 1).

**FIG 4:**
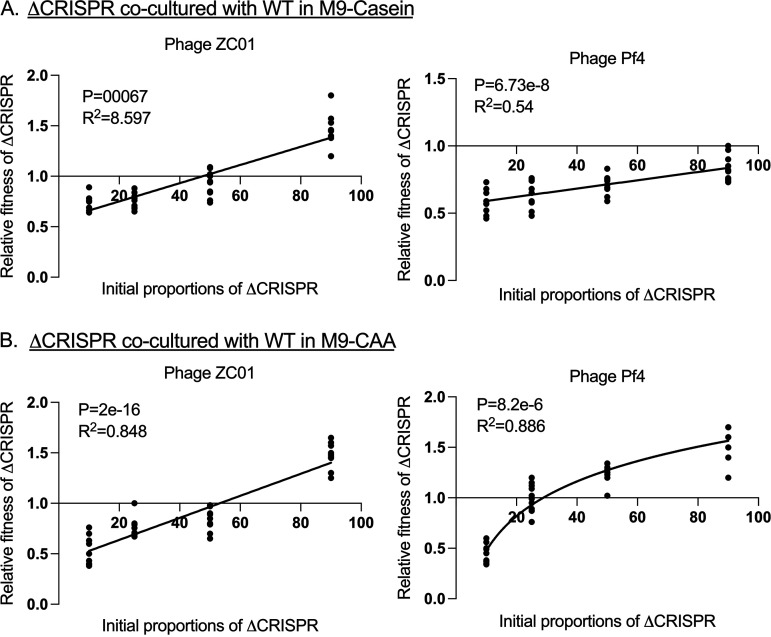
Frequency-dependent relative fitness of ΔCRISPR strains when exposed to phages. The relative fitness of ΔCRISPR strains cocultured with WT in M9-casein (A) and in M9-CAA (B) with the Pf4 and ZCO1 phages. The strains were cocultured for 24 h with their relative initial proportions as 10%, 50%, 75%, and 90% of the population. The goodness of fit is indicated by *R*^2^ and was analyzed using ANOVA with *P* values indicated (*n* = 10 biologically independent samples for each initial proportion of strains).

During ΔCRISPR coculture with the WT, exposure to the Pf4 and ZCO1 phages resulted in a positive frequency-dependent relative fitness of the ΔCRISPR strains in comparison to the WT in both M9-casein and M9-CAA (Fig. 4A and B). It is plausible that the relative fitness of the ΔCRISPR strains during competition with the WT depends on the initial proportions in the population as well as their response to specific phage infection. Although the ΔCRISPR strain shows altered production of some QS signals compared to the WT (Fig. S4), no significant difference was observed in the production of QS-regulated virulence factors such as pyocyanin, elastase, and protease (Fig. S5). The reduced fitness of the ΔCRISPR strains at <90% of the initial population in M9-casein may be due to their response to Pf4 infection rather than QS regulation.

10.1128/msystems.00092-22.5 FIG S4 Quorum-sensing signal production in the WT, ΔCRISPR and Δ*lasRrhlR* strains. (A to C) Signal production of 3OC12HSL (A), PQS (B), and C4HSL (C) in the P. aeruginosa strains after overnight culture in LB. The production of QS signals are normalized to the WT at 100%. Data are mean ± SD of *n* = 3 replicates. Download FIG S4, TIF file, 2.1 MB. Copyright © 2022 Ahator et al. 2022 Ahator et al. https://creativecommons.org/licenses/by/4.0/ This content is distributed under the terms of the Creative Commons Attribution 4.0 International license.

10.1128/msystems.00092-22.6 FIG S5 Virulence factor production. (A) Protease assay for P. aeruginosa strains. The assay was performed on LB supplemented with 1% skim milk. The diameter of the zone of clearance was measured. The figure is a representation of mean ± SD for *n* = 4 independent experiments. (B) Pyocyanin and elastase in P. aeruginosa strains grown overnight in LB at 37°C. The relative amounts of pyocyanin and elastase are expressed as a percentage of the wild type (WT). Data represent the mean ± SD of *n* = 4 experiments. Download FIG S5, TIF file, 2.1 MB. Copyright © 2022 Ahator et al. 2022 Ahator et al. https://creativecommons.org/licenses/by/4.0/ This content is distributed under the terms of the Creative Commons Attribution 4.0 International license.

**FIG 5:**
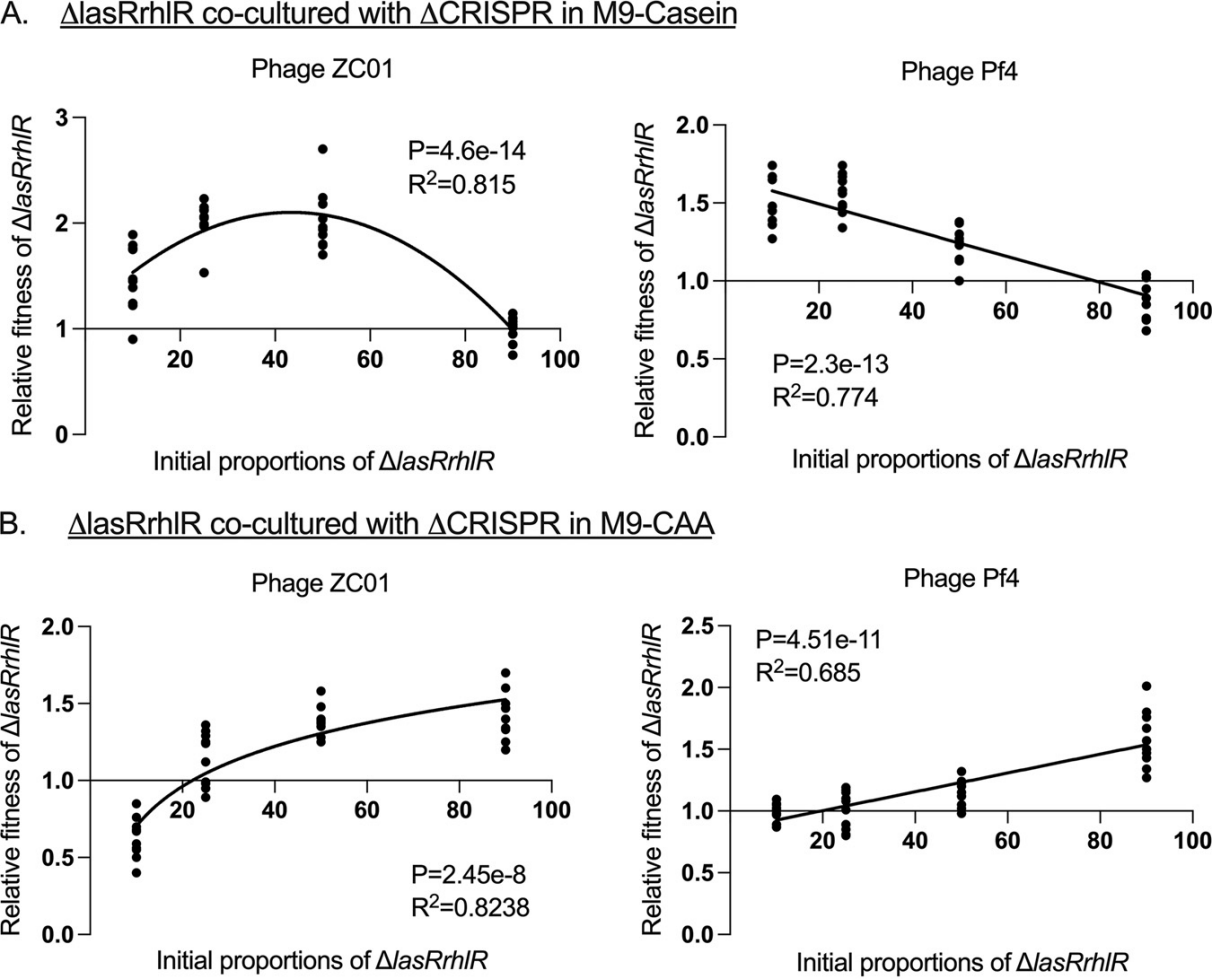
Frequency-dependent relative fitness of Δ*lasRrhlR* and ΔCRISPR strains when exposed to phages. The relative fitness of Δ*lasRrhlR* strains cocultured with ΔCRISPR in M9-casein (A) and in M9-CAA (B) with the Pf4 and ZCO1 phages. The strains were cocultured for 24 h with their relative initial proportions as 10%, 50%, 75%, and 90% of the population. The goodness of fit is indicated by *R*^2^ and was analyzed using ANOVA with *P* values indicated (*n* = 10 biologically independent samples for each initial proportion of strains).

From the cocultures of the Δ*lasRrhlR* with ΔCRISPR strains in M9-casein and M9-CAA, we observed that the frequency-dependent fitness of the Δ*lasRrhlR* strains in the population was influenced by factors such as their resistance to phages, reliance on the ΔCRISPR as cooperators for resources, and the initial proportion in the population. In M9-casein, Δ*lasRrhlR* shows a negative frequency-dependent relative fitness when cocultured with the ΔCRISPR strains. However, in M9-CAA, the Δ*lasRrhlR* strains exhibit a positive frequency-dependent relative fitness (Fig. 5A and B).

### QS- and CRISPR-mediated resistance and fitness are dependent on nutrient availability.

**FIG 6:**
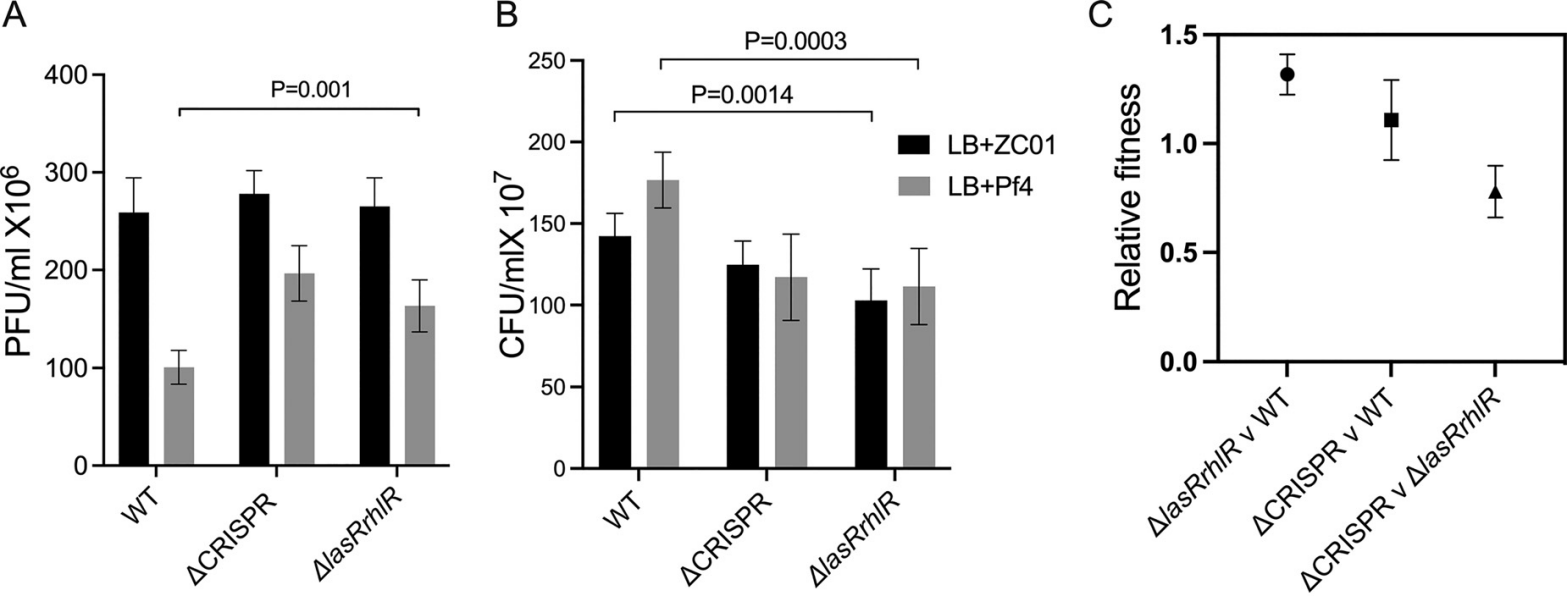
Phage infection capacity and fitness of P. aeruginosa strains grown in nutrient-rich conditions. (A) Pf4 and ZCO1 phage density was measured in P. aeruginosa strains grown in LB. The Pf4 and ZCO1 phages were added at the beginning of the experiment at a multiplicity of infection (MOI) of 0.001. (B) The corresponding bacterial densities following phage infection were measured by spread plating serial dilution of the culture on LB agar. The bacterial and phage numbers were determined after 16 h of phage infection. (C) The relative fitness of Δ*lasRrhlR*, ΔCRISPR, and WT strains competed in LB without phage exposure. Strains were mixed at equal proportions at the onset of the competition. Error bars represent ± SD of *n* = 5 independent experiments. A *P* value of <0.05 is considered significant.

Nutrient availability influences the bacterial-phage interactions and the QS-mediated social lifestyle of bacteria (11, 20, 34, 54). Considering the variations in phage infectivity and relative fitness of the WT, Δ*lasRrhlR*, and ΔCRISPR strains in the nutrient-limited M9 media, we decided to investigate their relative fitness and phage resistance in nutrient-rich Luria-Bertani (LB) medium. In the rich media, the Δ*lasRrhlR* strains were more susceptible to Pf4 than the WT (*t* = 4.848, degrees of freedom [df] = 8, *P* = 0.001), resulting in the increased phage accumulation (Fig. 6A and B). Also, the Pf4 phage density following infection of the ΔCRISPR strain increased significantly compared to the WT (*t* = 7.04, df = 8, *P* < 0.0001) (Fig. 6A and B). Thus, nutrient availability affects the response of the genetically diverse strains to phage exposure.

**FIG 7:**
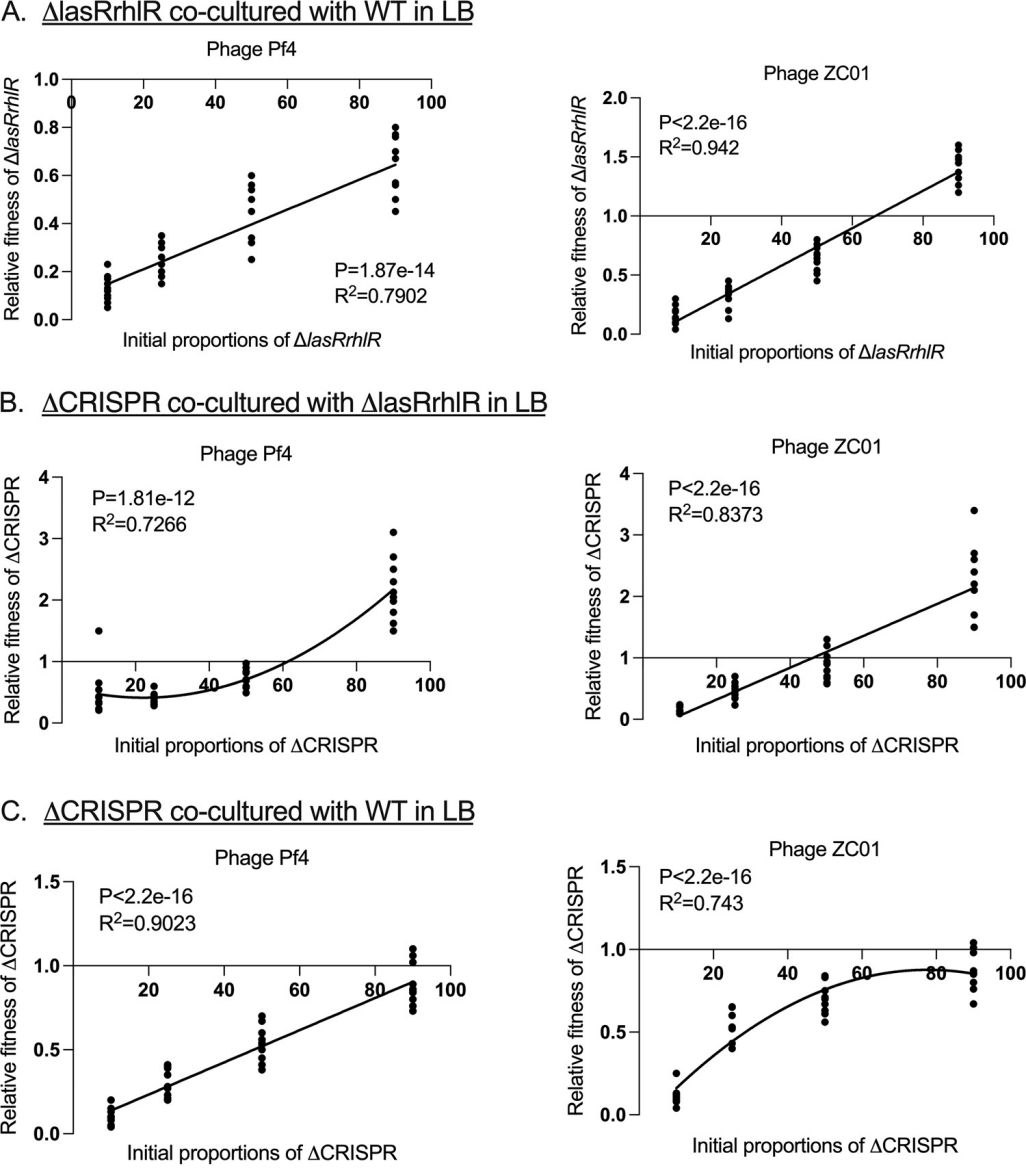
Relative fitness of P. aeruginosa strains during phage exposure in nutrient-rich conditions. (A to C) Relative fitness of Δ*lasRrhlR* strains cocultured with WT (A), ΔCRISPR strains cocultured with Δ*lasRrhlR* (B), and ΔCRISPR cocultured with the WT (C) in the presence of either Pf4 or ZCO1. The strains were cocultured for 24 h with their relative initial proportions as 10%, 50%, 75%, and 90% of the population. The goodness of fit is indicated by *R*^2^ and was analyzed using ANOVA with *P* values indicated (*n* = 10 biologically independent samples for each initial proportion of strains).

Next, we investigated the relative fitness of the strains in LB media in the absence of phage exposure. In the rich media, we believe that the increased fitness of the Δ*lasRrhlR* strain over the WT and ΔCRISPR strains is due to the growth advantage of the Δ*lasRrhlR* strain in LB (Fig. S2 and Fig. 6C). However, when exposed to phages, the Δ*lasRrhlR* strain showed reduced fitness compared to the WT (Δ*lasRrhlR*/WT, Pf4, *t* = 13.64, *P* < 0.0001; ZCO1, *t* = 4.82, *P* = 0.002) (Fig. 7A) and showed a positive frequency-dependent relative fitness when cocultured with the WT and ΔCRISPR strains (Fig. 7A and B). Several factors, such as the alleviation of the metabolic cost and growth advantage and phage resistance associated with loss of the CRISPR-Cas and QS systems, may account for the variations in relative fitness between the Δ*lasRrhlR* and the ΔCRISPR strains when cocultured in LB (34). We ruled out QS-dependent social cheating, as Δ*lasRrhlR* strains independently attained a higher growth rate and do not require QS-regulated exoproducts released by the ΔCRISPR strains in LB. Additionally, the ΔCRISPR strains showed reduced fitness when competed against the WT in the presence of phages except at high initial proportions in the population (Fig. 7C). The fitness advantage exhibited by the WT when competed against the Δ*lasRrhlR* and ΔCRISPR strains highlights the importance of the QS and CRISPR-Cas systems in mediating defense against Pf4 and ZCO1 in LB.

### Phage exposure and media conditions reshape bacteria community composition.

**FIG 8:**
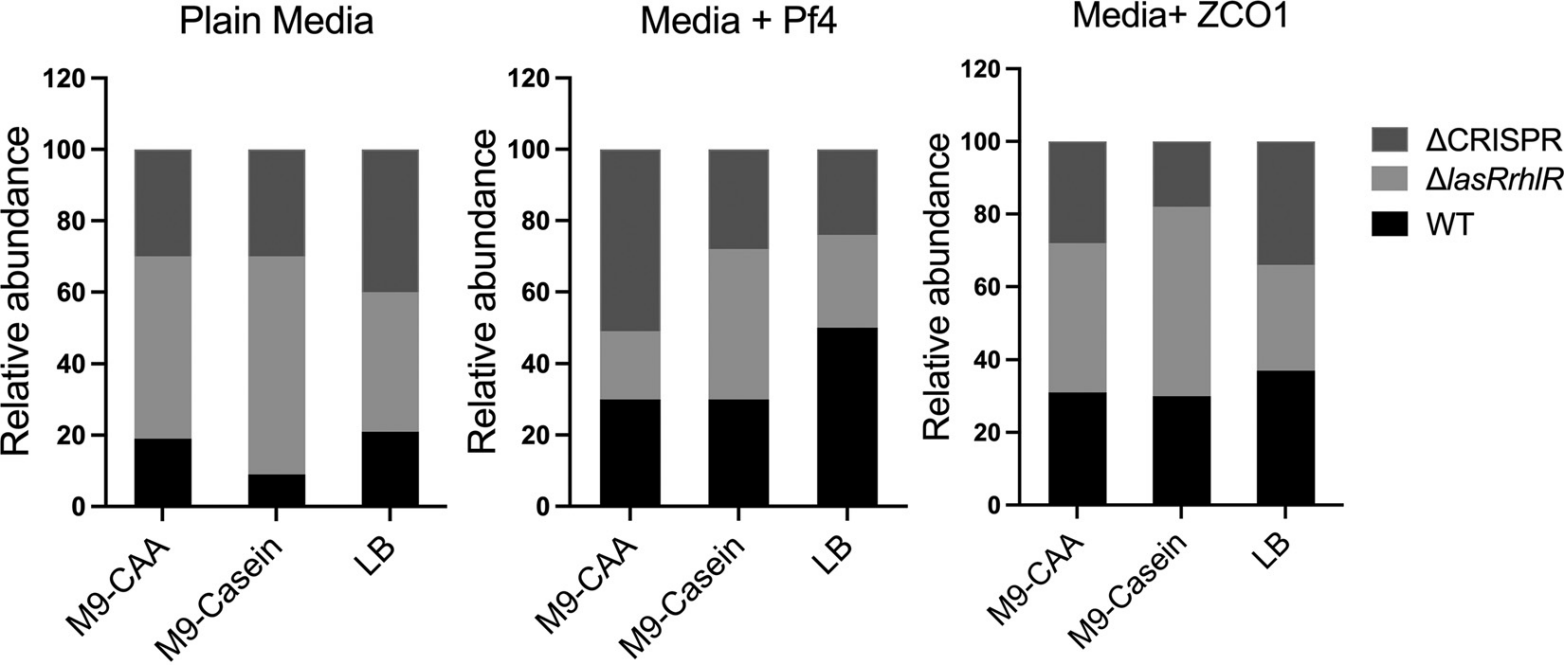
Phage exposure and nutrient availability alter the population dynamics of P. aeruginosa strains. P. aeruginosa community composition after 24 h of coculture in M9-CAA, M9-casein, and LB with and without phages. Equal proportions of the strains were mixed at the start of the experiment. The phages were added at an MOI of 0.001 at the start of the experiment. The cultures were incubated at 37°C at 150 rpm. The data represents the mean ± SD of 5 experiments.

To investigate the population dynamics of the P. aeruginosa strains when exposed to phages in both nutrient-rich and nutrient-limited conditions, we cocultured all three strains in LB, M9-CAA, and M9-casein media with and without phages and monitored the population dynamics of the strains after 24 h. All strains were added in equal proportions at the beginning of each coculture. In the absence of phages, the Δ*lasRrhlR* strains dominated the WT and ΔCRISPR strains when cocultured in nutrient-limited conditions (Δ*lasRrhlR* in M9-casein, ~51%, and M9-CAA, ~60% of the total bacterial population) (Fig. 8). However, when cocultured in LB without phages, we obtained relatively equal proportions of the Δ*lasRrhlR* and ΔCRISPR strains (~40%) from the cultures (Fig. 8). This shows that the fitness advantage associated with AHL-QS signal-blind mutants is more evident in nutrient-limited and QS media conditions than the nutrient-rich conditions.

During phage exposure in LB media, the WT was dominant in the population following the coculture with the Δ*lasRrhlR* and ΔCRISPR strains (WT-ZCO1, ~39%, and WT-Pf4, ~50% of the population) (Fig. 8).

We further quantified the phage densities obtained from the monocultures and cocultures at the end of the experiment (Fig. S6). In general, the phage densities obtained from the cocultures were lower than those obtained from the monocultures (Fig. S6). These results show that cooperation between the genetically diverse strains can improve phage resistance and enable maintenance of the social lifestyle of the population.

10.1128/msystems.00092-22.7 FIG S6 Phage density of PF4 and ZCO1 obtained from the 24-hour coculture and monocultures of the P. aeruginosa strains in M9-CAA, M9-casein, and LB. WT:Δ*lasRrhlR*:ΔCRISPR represents the coculture of the three strains. The data show the mean ± SD of 5 independent experiments. A *P* value of <0.05 is considered significant. Download FIG S6, TIF file, 2.1 MB. Copyright © 2022 Ahator et al. 2022 Ahator et al. https://creativecommons.org/licenses/by/4.0/ This content is distributed under the terms of the Creative Commons Attribution 4.0 International license.

## DISCUSSION

To understand the impact of phage exposure and nutrient availability on the cooperation and conflict between QS and CRISPR-Cas systems, we investigated the relative fitness of the AHL-QS signal-blind mutants and CRISPR-Cas mutants in P. aeruginosa when exposed to two different types of phages. Using rich and nutrient-limited media, we examined the effect of resource availability on the fitness and adaptation of the bacteria during phage exposure. In this study, we used the AHL signal-blind P. aeruginosa strains with *lasRrhlR* mutations, which show a fitness advantage over the QS-proficient strain and the signal-negative (*lasIrhlI* mutant) strain during coculture in media requiring QS exoproducts for breakdown of casein as a carbon source (12, 19). Also, clinical and environmental strains with defective or lacking CRISPR-Cas systems have been reported (35–37). Maintenance of the CRISPR system is metabolically costly. Hence, selection against the CRISPR-Cas system enhances the fitness advantage mostly in polymicrobial communities with minimal phage threat or in conditions where parasite exposure and nutrient availability do not favor CRISPR-Cas response (32, 34, 55) However, due to the abundance of phages exposed to bacteria in the human body (42, 47, 48, 56), selection against the Δ*lasRrhlR* and CRISPR-Cas mutants appears counterintuitive. Studies on bacterial phage infection models, social lifestyle, and bacterial adaptation have shown that bacterial adaptive response is partly dependent on the microbial community and environmental factors such as nutrient availability and the degree of parasite exposure (34, 41, 55). Based on the importance and interconnection between QS and CRISPR-Cas systems in responding to phage infections, we decided to investigate how nutrient availability and phage exposure influence P. aeruginosa population dynamics.

In this study, we observe the benefits of cooperation and conflict usually occurring among genetically diverse strains in microbial communities and how a mixed population of genetically diverse strains, which are common in nature (biofilms and clinical samples) (43, 46, 57, 58), can reshape the strategies of survival under various stress conditions. Sharing public goods between cooperators and cheats is beneficial for survival under nutrient-limited conditions. Also, the reduction in virulence factor productions observed in the cheats is a hallmark of persistence and ability to evade detection by host immune responses. Additionally, the reduction in phage density (Fig. S6) following infection in the mixed-culture competition assay (Fig. 8) shows the possible benefits of evolving resistance due to diversity in a microbial population. Further investigations into the evolution of phage resistance among strains in the competition assay will provide more insight into the underlying mechanisms driving the population dynamics among genetically diverse bacteria. Understanding the evolutionary mechanism for the maintenance of cooperation or conflict in microbial communities can have a potential impact on controlling microbial infections and applications in systems biology.

Our work supports previous findings that the growth rate and high population density of bacteria predispose them to phage infection (25, 27, 50). We showed that the AHL-QS signal-blind mutants exhibit high phage resistance due to the reduced growth rate and low cell density in the M9-casein medium.

The signal-blind cheats are known to dominate the population of P. aeruginosa strains by avoiding the metabolic cost of producing extracellular factors encoded by QS-activated genes but exploit those released into the environment by members of the population. Growth in M9-casein promotes social cheating and the emergence of cheats in the bacterial community where QS-regulated factors such as proteases are necessary for growth (20). In the absence of phages, when cocultured at equal proportions, the signal-blind cheats gain a higher competitive advantage over the WT compared to during phage exposure (Fig. 2 and 3). This shows that phage exposure influences the community lifestyle of the P. aeruginosa population (41). Apart from the exploitation of public goods by the cheats, since the selective advantage of QS signal-blind cheats is dependent on the proportion of cooperators present in the population (12, 15), the reduction in fitness advantage of the signal-blind cheats can also be attributed to the decline in the WT and ΔCRISPR strain (cooperator) population due to phage infection. On the other hand, in rich media where Δ*lasRrhlR* mutants do not require QS-regulated exoproducts for growth, they show lower fitness than the WT when exposed to phages. Also, the increased susceptibility of the Δ*lasRrhlR* to phage infection further contributes to the decrease in fitness.

Based on the social evolution theory, the fitness of cheats such as Δ*lasRrhlR* strains in a microbial population is frequency dependent and decreases as their population density increases, resulting in overexploitation of the cooperators (20, 53). The Δ*lasRrhlR* mutants exhibited a negative frequency dependence when competed against the WT and ΔCRISPR strains in M9-casein in the presence of both phages. Thus, in M9-casein, the fitness advantage of the Δ*lasRrhlR* strains increases as the initial proportion of the WT and ΔCRISPR strains increases. However, the decline in the WT and ΔCRISPR strain population due to phage infections results in fewer cooperators maintaining the production of public goods required by the Δ*lasRrhlR* strains (Fig. 3 and 5). As the Δ*lasRrhlR* strains can grow in M9-CAA media independent of the public goods produced by the WT and ΔCRISPR strains, their relative fitness is therefore dependent on their resistance to phage infection and initial frequency in the population.

Competition between the ΔCRISPR strain and the WT revealed that their relative fitness is influenced by their individual response to phage exposure. The increased fitness of the ΔCRISPR strain over the WT when cocultured in M9-casein in the absence of the Pf4 and ZCO1 phages could be due to their relative growth rate (Fig. S2) or the altered QS signal production (Fig. S4). However, we ruled out the effect of metabolic cost associated with the production of QS-regulated factors such as pyocyanin, elastase, and protease as the underlying cause for the variations in fitness between the WT and ΔCRISPR strains (12, 16, 59) since there was no significant difference in the production of these factors among the WT and ΔCRISPR strains (Fig. S5). Also, loss of the CRISPR-Cas system is shown to alleviate the metabolic burden associated with its maintenance and expression (32, 34).

The evolution of phage resistance varies depending on the type of phage and its mode of infection in bacteria (60). From our study, we observed that the susceptibility of the strains, relative fitness, and population dynamics in different nutrient resources differ when exposed to the two phages. The filamentous pseudomonas phage Pf4 with 12-Kbp single-stranded DNA (ssDNA) genome architecture is a member of the *Inoviridae* with the ability to convert from a lysogenic phage to a superinfection variant depending on environmental cues (61). The Pf4 phage infection enhances filamentation and extracellular DNA (eDNA) release by P. aeruginosa, which favors biofilm formation and resistance to antimicrobials (62). The Pf4 phage has the ability to infect and form plaques on P. aeruginosa without integrating into the genome (63). Also, Pf4 is suggested to target type IV pili in P. aeruginosa (58). However, since we did not assay for lysogeny or test for pili attachment by the phage in our study, we are unable to emphatically state the resistance mechanism deployed by the strains when exposed to Pf4. Further investigations are therefore required to understand the mechanisms employed by the CRISPR-Cas and QS systems in defending against Pf4 infection. The phage ZCO1 belonging to the *Siphoviridae* YuA-like viral genus has a narrow host range and forms clear plagues typical of lytic phages on P. aeruginosa (64). Unlike Pf4, the ZCO1 phage reduces biofilm cell densities of P. aeruginosa PA14 and increases their susceptibility to antibiotics (64). The observed differences in phage infection capacity, the fitness of strains, and population dynamics observed in this study may differ in some sense from those seen in other studies (21, 34, 41, 65) considering the medium compositions and type of phages used. Other P. aeruginosa strains such as PAO1, which is QS proficient but less virulent and lacks the type I-F CRISPR-Cas system, may demonstrate contrasting dynamics when exposed to these phages.

This study relied on the presence and absence of the genes required for AHL-QS regulation and the CRISPR-Cas system to study the impact of both systems on population dynamics during phage exposure under different nutrient conditions. Further experiments investigating the lysogeny or spacer acquisition into the CRISPR arrays will be required to ascertain the molecular mechanism of the bacterial resistance to the phages used in this study. During the 24-h incubation period used for these coculture experiments, we did not observe a complete loss of a particular strain during the coculture in all the conditions tested. It is possible that the complete disappearance of one or two strains or the collapse of the sociomicrobiology of the strains, termed tragedy of the commons, may be observed in extended incubation periods of the cocultures. The 24-h coculture was long enough to investigate the effects of the nutrient availability and phage exposure on the bacterial population dynamics, as prolonged cultures without subsequent passage into fresh media will result in nutrient depletion and the introduction of other stress factors into the experimental setup.

The development of phage therapy will require an understanding of the coevolutionary dynamics between bacteria and phages. Due to the diversity of phages, it is empirical to investigate their dynamic interaction with their hosts in the context of the host environment. Such knowledge of the relationship between phage infection, bacterial immune response, bacterial community lifestyle, and the influence of abiotic factors on bacterial-phage interactions will highlight key strategies in developing phage therapy.

## MATERIALS AND METHODS

### Strains, phages, vectors, and medium conditions.

The P. aeruginosa UCBPP-PA14 strain, designated PA14, was used as the wild type (WT) for generating the CRISPR-Cas mutant (ΔCRISPR) and the AHL-QS signal-blind mutant (Δ*lasRrhlR*) for the experiments. The phages used in the experiment were extracted from P. aeruginosa strains. The lysogenic filamentous phage Pf4 was provided by Scott Rice (Nanyang Technological University), Singapore (61). Also, the lytic P. aeruginosa phage ZCO1 of the family *Siphoviridae* from our lab collection was used in this study.

The Luria-Bertani (LB) and M9 minimal medium (M9) (Na_2_HPO_4_·7H_2_O, 12.8 g; KH_2_PO_4_, 3 g; NaCl, 0.5 g; NH_4_Cl, 1.0 g; 1 M MgSO_4_, 200 μL; 1 M CaCl_2_, 10 μL; and deionized H_2_O, up to 1 L) supplemented with 1% Caseinate (M9-casein) or 0.5% Casamino Acids (M9-CAA) (19, 66) as a carbon source were used for phage infectivity and competition assay of the strains. The M9-casein and M9-CAA were used as nutrient-limited conditions, where M9-casein (QS media) induces QS behavior and promotes social cheating.

### Design and cloning of vectors.

The open reading frames of EGFP and mCherry were cloned using primers given in Table S1 in the supplemental material and inserted into the multiple-cloning site of the Pseudomonas shuttle vector pUCP19. The vector was then transformed into Escherichia coli and spread on LB supplemented with carbenicillin (200 mg/mL) and 50 μg/mL of X-Gal (5-bromo-4-chloro-3-indolyl-β-d-galactopyranoside). The E. coli transformants with the correct constructs were selected by PCR and DNA sequencing. The correct constructs were purified and transformed into the P. aeruginosa strains. The P. aeruginosa transformants were selected on LB agar supplemented with carbenicillin (300 mg/mL) and 50 μg/mL of X-Gal. The expression and verification of the fluorescence vectors were performed by fluorescence microscopy and fluorescent spectroscopy. The expression of the *lacZ* was verified with the beta-galactosidase assay and blue/white screening on LB agar media supplemented with 50 μg/mL of X-Gal.

10.1128/msystems.00092-22.1 TABLE S1 Primers for in-frame deletion and fluorescent reporter constructs. Download Table S1, XLSX file, 0.01 MB. Copyright © 2022 Ahator et al. 2022 Ahator et al. https://creativecommons.org/licenses/by/4.0/ This content is distributed under the terms of the Creative Commons Attribution 4.0 International license.

### Deletion of QS and CRISPR-Cas genes.

The in-frame deletion of the *lasR*, *rhlR*, and the genes comprising the type I-F CRISPR-Cas in the PA14 was performed using the list of primers given in Table S1. The DNA fragment flanking the region of the *lasR* and *rhlR* genes was ligated with the linearized vector pK18mobsacB and transformed into E. coli DH5α for selection of constructs with the correct orientation of the up and down fragments. The correct constructs were transformed into E. coli S17-1 for conjugation with PA14. The transconjugants were selected on minimal medium (MM; each L contains mannitol, 2.0 g; (NH_4_)_2_SO_4_, 2.0 g; K_2_HPO_4_, 10.5 g; KH_2_PO_4_, 4.5 g; MgSO_4_·7H_2_O, 2.0 g; FeSO_4_, 5 mg; CaCl_2_, 10 mg; and MnCl_2_, 2 mg; pH 7.0) supplemented with gentamicin (30 μg/mL) and point inoculated onto MM supplemented with 10% (wt/vol) sucrose to select mutants. Mutants containing the desired deletion were verified by PCR and DNA sequencing.

### Competition assay.

The competitive ability of the strains was measured by growing all strains in 10 mL of the specified media for 24 h. Strains were marked by expressing EGFP, mCherry, and LacZ in *trans* from a pUCP19 vector backbone. Overnight LB-MOPS (morpholinepropanesulfonic acid) broth cultures were inoculated into the media for the competition assay. We measured the competitive ability in cocultures of P. aeruginosa strains in LB, M9-CAA, and M9-casein media. The media were supplemented with carbenicillin (300 μg/mL) to maintain vector expression during the competition assay. Overnight cultures in LB-MOPS were normalized to approximately 10^8^ CFU/mL and used for inoculation in the specified media. Both Pf4 and ZCO1 phages (3 × 10^6^ PFU/mL) were added at the start of the experiment. For all monocultures and coculture or mixed-culture infections with Pf4 and ZCO1, we maintained a final multiplicity of infection (MOI) of 0.001 to prevent differences in phage or bacterial densities during the infection. The mixed cultures were allowed to grow for 24 h at 37°C with shaking. Briefly, 300 μL of each coculture was sampled at time of 0 h (initial frequency) and time of 24 h (final frequency), and aliquots from serial dilutions were plated on LB supplemented with carbenicillin (300 μg/mL) and/or X-Gal (for blue/white screening) to determine cell yield and calculate CFU. Additional confirmation of Δ*lasRrhlR* mutants was performed by point inoculating colonies on protease assay plate (LB with 1% skim milk) and detection of fluorescence using a fluorescent microplate reader.

The fitness of each strain in the coculture was calculated by comparing their initial and final frequencies during the experiment (12, 53). The initial frequency was calculated by counting the number of colonies at the start of the experiment at time of 0 h. The final frequency was calculated by counting the number of colonies at the end of the experiment, where time is 24 h. For two strains, *X* and *Y*, the relative fitness of strain *X* is relative fitness = *X*_1_(1 − *X*_0_)/*X*_0_(1 − *X*_1_), where *X*_0_ and *X*_1_ are the initial and final frequencies of strain *X*, respectively. Relative fitness of >1 implies that strain *X* increases in frequency over strain *Y*. Relative fitness of 1 means they remain at the same frequency. Relative fitness of <1 indicates strain *X* decreases in frequency compared to strain *Y* (20).

### Phage infection assay (plaque assay) and colony count.

Estimation of the susceptibility of the P. aeruginosa strains and infectivity of the phages was performed using the spread plate method and double agar layer method, respectively. The CFU of the P. aeruginosa strains was measured by collecting 1 mL of culture after the experiment and spread plating an aliquot of the serial dilution onto LB agar. The plates were incubated overnight for at least 16 h at 37°C, after which the colonies were counted.

The phage plaque assay was performed using the double agar layer method. Four milliliters of the infected P. aeruginosa cultures were collected at the end of the experiment. The cells were centrifuged at 12,000 rpm for 5 min to obtain the supernatant. The phages were extracted using chloroform, and the cell-free supernatant was filtered through a 0.2-mm syringe filter into a sterile 15-mL Falcon tube. The phage extracts were serially diluted, and 200 μL combined with 300 μL of the P. aeruginosa WT was grown to an optical density at 600 nm (OD_600_) of 0.5. This suspension of bacteria and phage was mixed with the top layer (0.75%) of LB agar poured onto the bottom agar and allowed to set. The plates were incubated at 37°C overnight to observe and quantify the plaques formed.

### Virulence factor quantification.

Pyocyanin was assayed from P. aeruginosa strains cultured in LB medium for 16 h at 37°C and shaken at 250 rpm. The cultures were centrifuged at 12,000 rpm for 5 min to obtain cell-free supernatants, which were mixed with an equal volume of chloroform and shaken for 30 min at room temperature. The solvent phase was mixed with 5 mL 0.2 N HCl and rocked at room temperature for 30 min followed by quantification of pyocyanin by measuring the absorbance of the supernatant at 520 nm (*A*_520_) and normalizing against the cell density at OD_600_.

Elastase production was quantified using the elastin-Congo red (ECR) (Sigma) assay (8). P. aeruginosa strains were cultured in LB for 16 h at 37°C and shaken at 250 rpm. The cultures were centrifuged at 12,000 rpm for 5 min to obtain cell-free supernatant, and 500 μL of cell-free supernatant was mixed with an equal volume of 5 mg/mL elastin-Congo red with ECR buffer. The mixture was incubated at 37°C with agitation for 2 h. The intensity of Congo red dye released from the elastin digestion is indicative of the amount of elastase in the supernatant, and this was quantified by spectrophotometry at *A*_520_ and normalized against the cell density at OD_600_.

Quantification of protease production was performed by inoculating a single colony of P. aeruginosa strains on LB agar supplemented with 1% skim milk. Casein hydrolysis observed as a clear zone around the bacterial colony is indicative of protease production (67). The diameter of the clear zone was measured.

### Quorum-sensing signal quantification.

QS signals were extracted from P. aeruginosa cells grown overnight in 5 mL LB broth at 37°C and shaken at 250 rpm. The signals were extracted with an equal volume of acidified ethyl acetate (0.1% acetic acid) twice from the cell-free supernatant. The organic phase (acidified ethyl acetate) was dried with nitrogen gas, and the dried extract was dissolved in 1 mL filtered high-performance liquid chromatography (HPLC)-grade methanol for liquid chromatography-mass spectrometry (LC-MS) analysis.

HPLC was performed on a Dionex UltiMate 3000 system (Thermo Fisher Scientific) using a C_18_ reverse-phase column (Thermo Fisher Scientific) with various methanol gradients and 0.1% acidified water as the mobile phase. The gradient profile for chromatography was 2% methanol for 1.5 min, linear increase in methanol to 100% over 5 min, 100% methanol for 4 min, and equilibration with 2% methanol and 98% water for 1.5 min. The flow rate was 0.4 mL/min.

Signal detection was performed with electrospray ionization coupled with high-resolution mass spectroscopy (HESI-MS; Q Exactive Focus; Thermo Fisher Scientific). The analysis was performed under positive ionization mode. The ion source settings were auxiliary gas flow rate of 10 (arbituary units), sheath gas flow rate of 40, sweep gas flow rate of 0, spray voltage of 4 kV, 320°C capillary temperature, 350°C heater temperature, and S-lens RF level of 50. Nitrogen was used as a nebulizing gas by the ion trap source. The MS/MS method was designed to perform an MS1 full-scan (100 to 1,000 *m/z*, no fragmentation) together with the SIM model. Settings for the SIM method were resolution of 35000 (full width half maximum [FWHM]) at 1.0 *m/z* isolation offset, and isolation window and centroid spectrum of 4.0. Signal mass scans were set for 3OC12-HSL at 298.20128 *m/z*, C4-HSL at 172.09682 *m/z*, and Pseudomonas quinolone signal (PQS) at 260.1645 *m/z*. Data analysis was performed using Thermo Xcalibur software (Thermo Fisher Scientific) and TraceFinder (Thermo Fisher Scientific).

### Growth curve.

The growth curve of the strains was measured using the Bioscreen C MBR. Starter cultures of the P. aeruginosa strains were inoculated into the specified sterile media loaded in the 100-well plate for the analyzer. The growth of the strains was monitored by measuring the absorbance at 600 nm every 15 min for 2 to 3 days at 37°C with normal speed and medium amplitude. The growth assay was conducted in triplicate. The data obtained were analyzed and plotted with GraphPad Prism.

### Statistical analysis.

Graphical presentation and statistical analysis were performed using R and GraphPad Prism.
