# Supplementary figures and images for: Nutrient Availability and Phage Exposure Alter the Quorum-Sensing and CRISPR-Cas-Controlled Population Dynamics of Pseudomonas aeruginosa
Source: mSystems. 2022 Jun 14;7(4):e00092-22. doi: 10.1128/msystems.00092-22 (PMC9426516; doi:10.1128/msystems.00092-22)

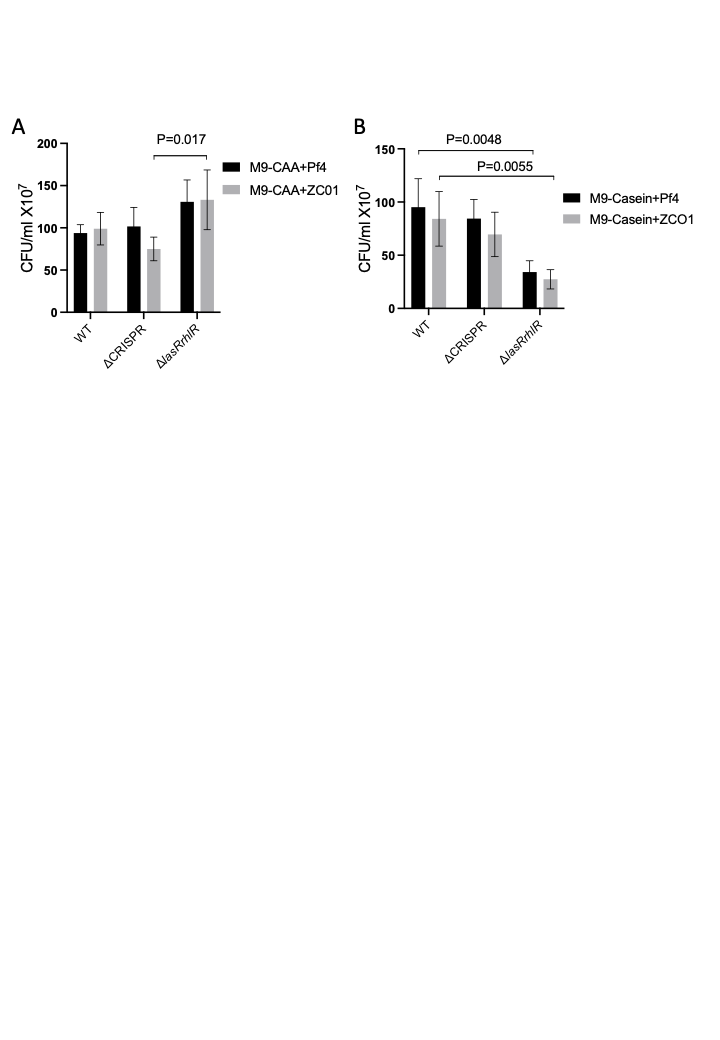

Supplement: FIG S1 [file msystems.00092-22-s0002.tif]

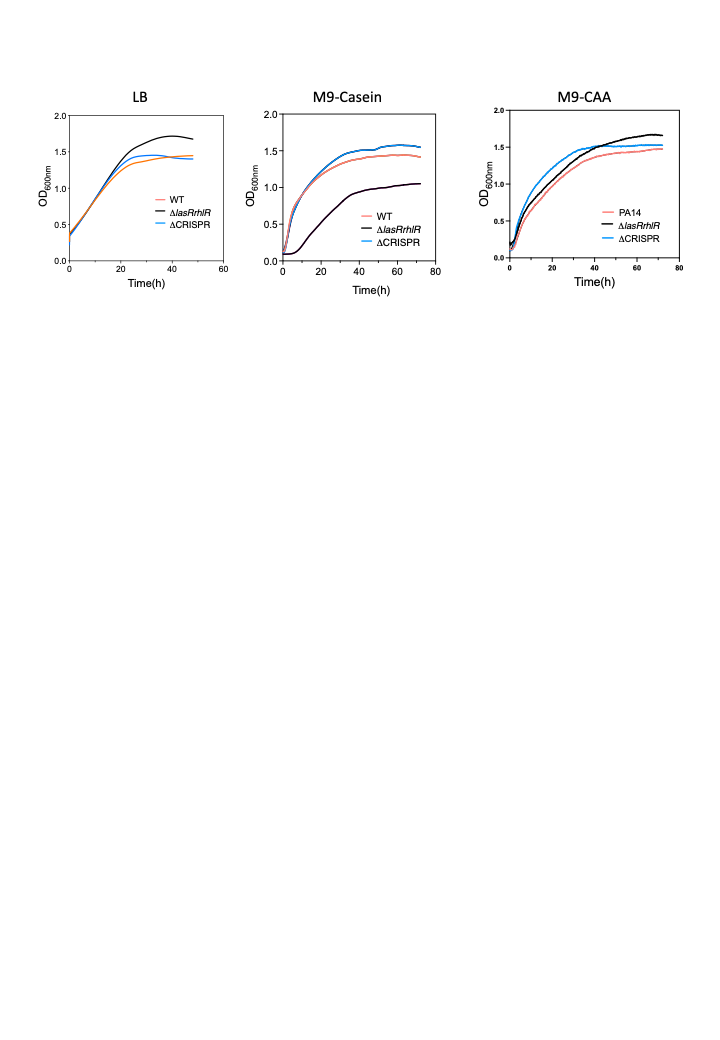

Supplement: FIG S2 [file msystems.00092-22-s0003.tif]

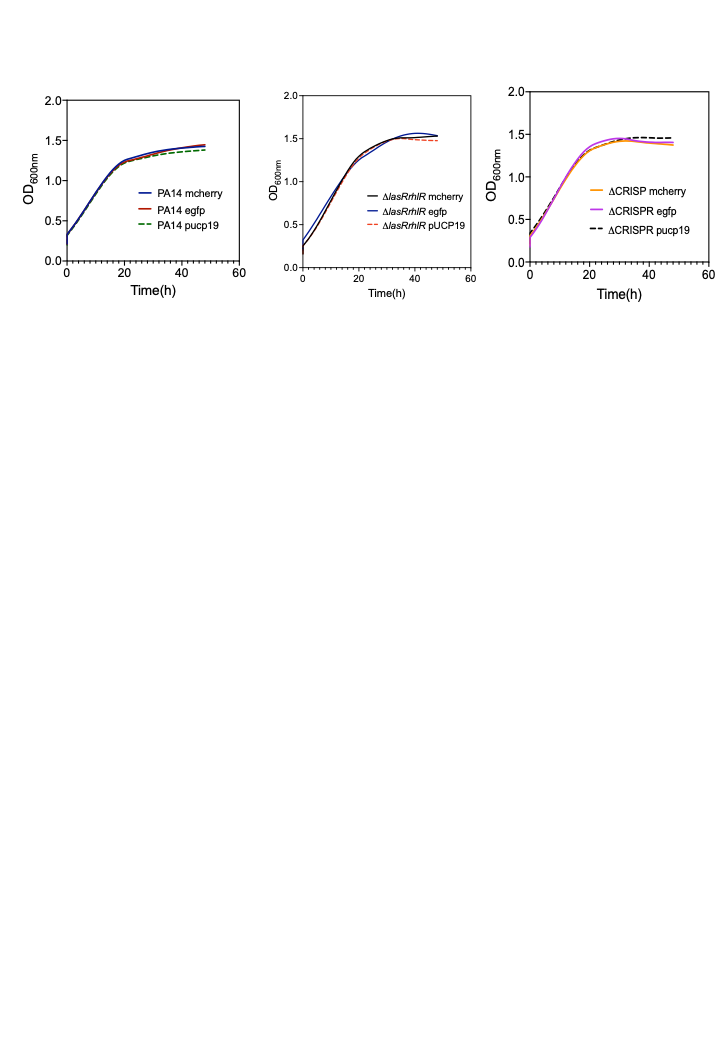

Supplement: FIG S3 [file msystems.00092-22-s0004.tif]

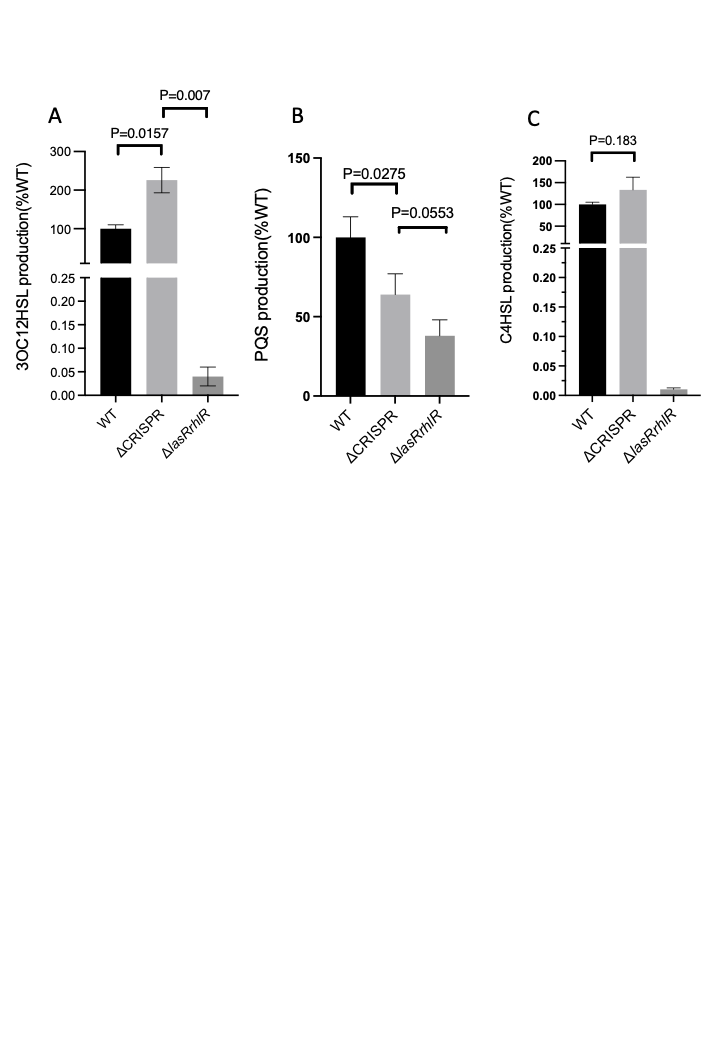

Supplement: FIG S4 [file msystems.00092-22-s0005.tif]

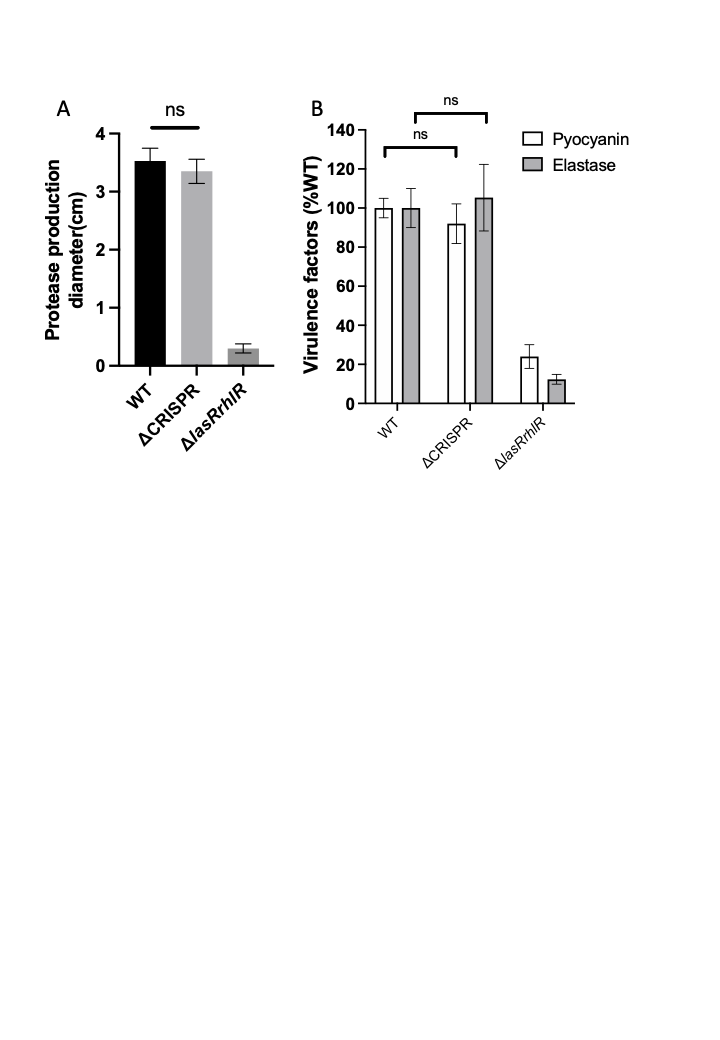

Supplement: FIG S5 [file msystems.00092-22-s0006.tif]

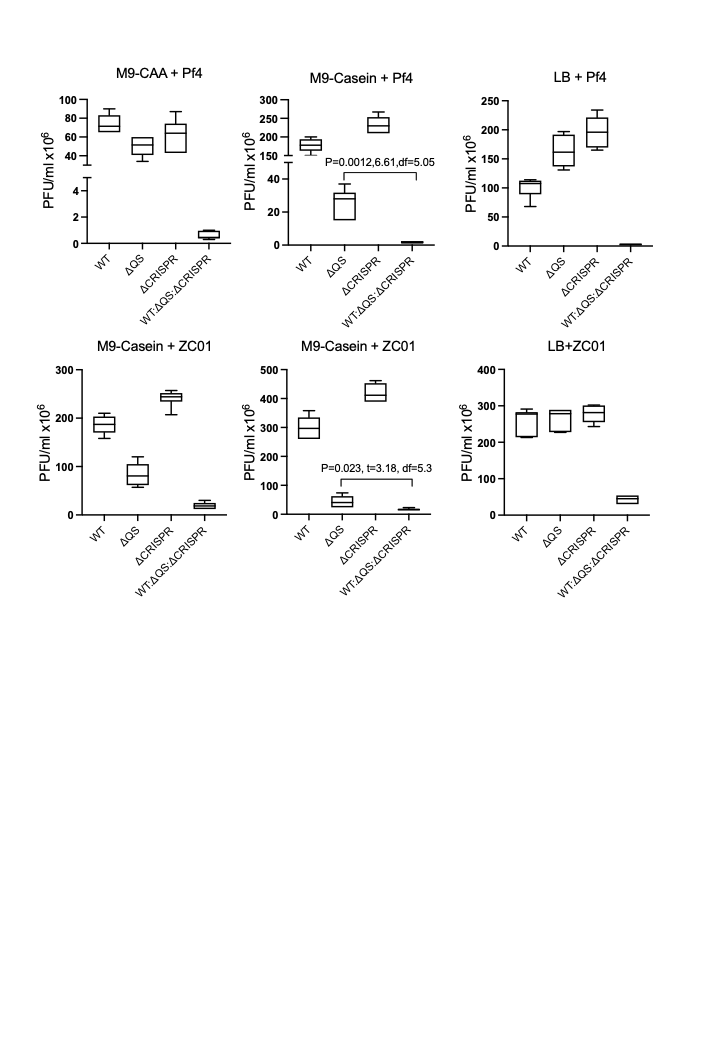

Supplement: FIG S6 [file msystems.00092-22-s0007.tif]
